# Flexible fibreoptic intubation in swine – improvement for resident training and animal safety alike

**DOI:** 10.1186/s12871-020-01127-2

**Published:** 2020-08-17

**Authors:** Robert Ruemmler, Alexander Ziebart, Thomas Ott, Dagmar Dirvonskis, Erik Kristoffer Hartmann

**Affiliations:** Department of Anaesthesiology, Medical Centre of the Johannes Gutenberg-University, Langenbeckstrasse 1, 55131 Mainz, Germany

**Keywords:** Airway management, Animal safety, Difficult airway, Fibreoptic intubation, Resident training

## Abstract

**Background:**

Efficient airway management to facilitate tracheal intubation encompasses essential skills in anaesthesiologic and intensive care. The application of flexible fibreoptic intubation in patients with difficult airways has been identified as the recommended method in various international guidelines. However, providing the opportunity to adequately train residents can be challenging. Using large animals for practice during ongoing studies could help to improve this situation, but there is no recent data on fibreoptic intubation in swine available.

**Methods:**

Thirty male German landrace pigs were anesthetized, instrumented and randomized into two groups. The animals were either intubated conventionally using direct laryngoscopy or a single-use flexible video-endoscope. The intervention was carried out by providers with 3 months experience in conventional intubation of pigs and a brief introduction into endoscopy. Intubation attempts were supervised and aborted, when SpO2 dropped below 93%. After three failed attempts, an experienced supervisor intervened and performed the intubation. Intubation times and attempts were recorded and analysed.

**Results:**

Flexible fibreoptic intubation showed a significantly higher success rate in first attempt endotracheal tube placement (75% vs. 47%) with less attempts overall (1.3 ± 0.6 vs. 2.1 ± 1.3, *P* = 0.043). Conventional intubation was faster (42 s ± 6 s vs. 67 s ± 10s, *P* < 0.001), but showed a higher complication rate and more desaturation episodes during the trial.

**Conclusions:**

Flexible fibreoptic intubation in swine is feasible and appears to be a safer and more accessible method for inexperienced users to learn. This could not only improve resident training options in hospitals with animal research facilities but might also prevent airway complications and needless animal suffering.

## Background

The efficient and safe airway management is one of the most important and outcome-relevant anaesthesiologic skills and directly affects perioperative mortality [1–4]. Flexible fibreoptic intubation (FFI) is recommended in national guidelines in the condition of an anticipated difficult airway [5–7] as well as a method amongst others in the unanticipated difficult airway [3, 5, 6] in order to safely facilitate tracheal tube placement in humans.

By comparison, while actual lung physiology is similar to humans and is regularly used for translational research in anaesthesiologic experiments [8], the airway management of swine can be challenging due to special anatomic properties on the orotracheal level [9–11]. The rate of encountered airway problems, resulting mortality or negative experimental effects in swine has not been sufficiently reported but is frequently mentioned [12–14]. Accordingly, in terms of clinical assessment, swine may inhere an anticipated difficult airway, thus warranting the search for alternative strategies to secure tracheal tube placement. Subsequently, FFI could be a promising option to preserve positive trial outcomes without unnecessary animal losses.

However, the adequate establishment and maintenance of crucial technical skills concerning FFI often poses organisational and structural problems in the reality of clinical practice and hospital environments [15, 16]. Although the use of large animal models has been suggested and was rated superior to manikin-based simulations [17], no further prospective examinations in the field have been conducted since and porcine models are not implemented in standard training protocols to date.

In this prospective randomized pilot trial, we evaluated the feasibility and effectiveness of video-enhanced FFI by inexperienced providers in swine compared to conventional intubation (CI), while simultaneously assessing complication rates during orotracheal intubation. We hypothesized that the use of FFI would show a higher success rate and therefore provide a safer tracheal access than direct laryngoscopy. Specifically, we assessed intubation attempts necessary as well as the time needed to successfully secure the airway. Additionally, we discuss whether porcine models can be used as a training tool for FFI and the maintenance of proficiency in this technique.

## Methods

### Anaesthesia

The study was approved by the State and Institutional Animal Care Committee (Landesuntersuchungsamt Rheinland-Pfalz, Koblenz, Germany, approval no. G16–1-042) with an additional approval (Issue date: 8/28/2019) for the dual use of the animals in this protocol. Thirty male German landrace pigs (12–16 weeks, 28-35 kg) were acquired from a local farm and received pre-transport sedation via an intramuscular injection of azaperone (2 mg kg^− 1^) and ketamine (4 mg kg^− 1^). Once in our Large Animal Research Facility, anaesthesia was induced via an ear cannula (22G) by injecting fentanyl (4 μg kg^− 1^), propofol (4 mg kg^− 1^) and atracurium (0.5 mg kg^− 1^) as described before [18]. During the whole experiment, anaesthesia was maintained via continuous infusion of propofol (5-10 mg kg^− 1^ h^− 1^) and fentanyl (8-12 μg kg^− 1^ h^− 1^) as well as a balanced electrolyte infusion (5 ml kg^− 1^ h^− 1^). The animals were transferred into a supine position and mechanically ventilated with a custom ventilation nose cone [19] using an intensive care respirator (Engstroem care station, GE healthcare, Munich; tidal volume 6-8 ml/kg, peak inspiratory pressure of 40cmH_2_O, positive end expiratory pressure of 5cmH_2_O). Adequate ventilation was confirmed by capnography, peripheral oxygen saturation and auscultation of the thorax.

### Intervention/ measurements

**Fig. 1:**
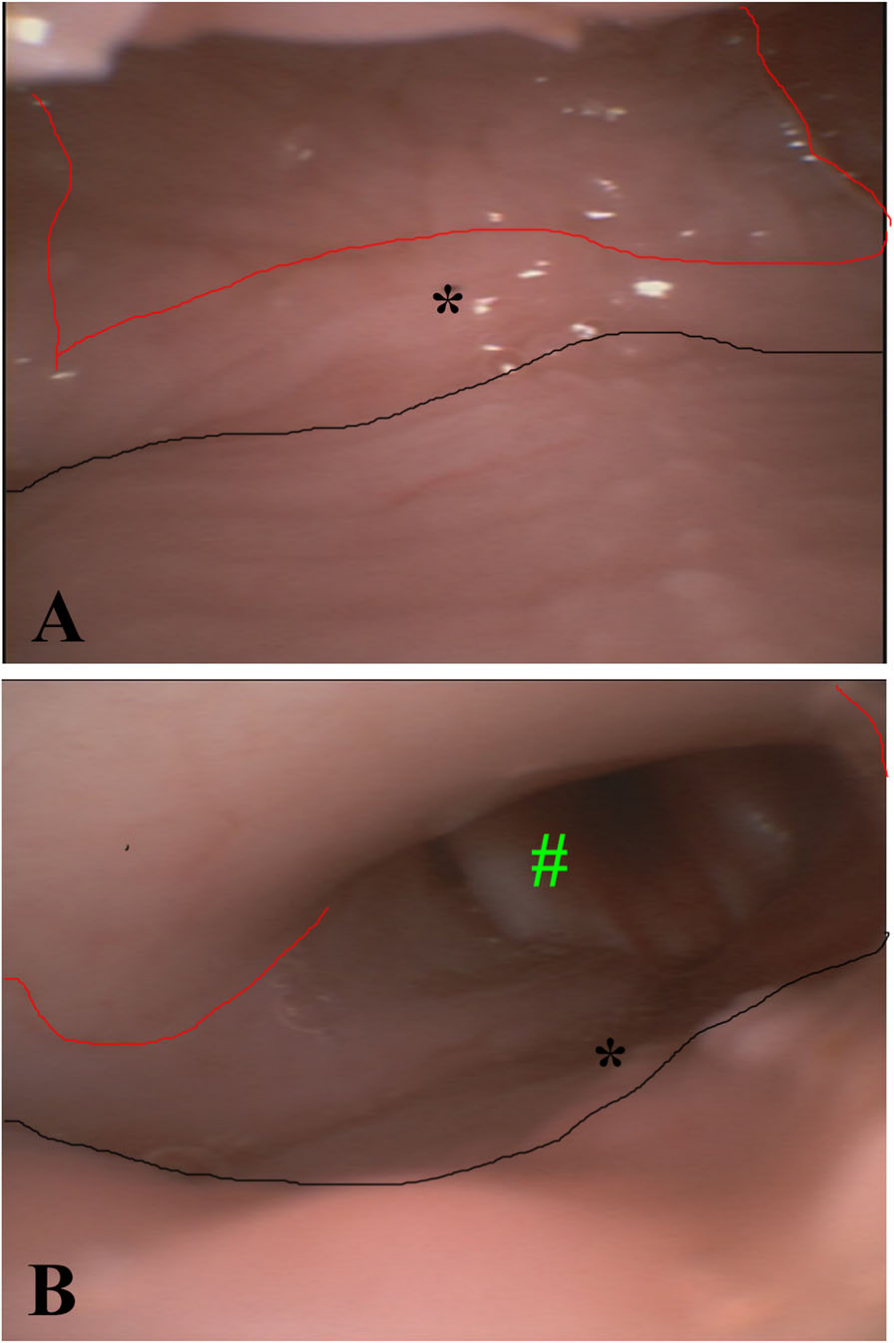
Porcine laryngeal anatomy in supine position before (**a**) and after mobilisation of the epiglottis (**b**). Before mobilisation, the epiglottis (red line) is fixated behind transitional tissue between the hard palate (black line) and the soft palate (*). After mobilisation, the epiglottis usually stays slightly ventrally dislodged and the larynx (green #) can easily be identified

After 4 min of mask ventilation, the epiglottis of the swine was mobilized from the soft palate by the supervisor (Fig. 1) using a Macintosh blade (size 4) as well as a tube guiding rod and performing a careful scooping motion from the right piriform recess to the left along the soft palate. After the dislodgement was visually confirmed, the animals were randomized into two groups:

CI group (conventional intubation): Animals were intubated via direct laryngoscopy using a MacIntosh blade (size #4, large) and a standard tracheal tube (TT, internal diameter (ID) 7.0 mm).

FFI group (flexible fibreoptic intubation): Animals were intubated using a single-use endoscope with video monitoring (Ambu aScope regular and Ambu aView, Ambu GmbH, Bad Nauheim, Germany) using a standard TT (ID 7.0 mm).

An exemplary video of FFI taken during the trial is available in the supplemental material of this article. As participants we used 3rd year medical students who had undergone basic training in animal handling in the university’s Translational Animal Research Center and were approved by the State and Institutional Animal Care Committee. The participants had been introduced to large animal experiments and conventional intubation (mean of performed CIs: 4.5) for 3 months prior to the trial. A short explanation and standardized introduction to the FFI device was performed by the supervisor before the first use. None of the participants had used a flexible video-endoscope before or seen a FFI over a video monitor.

Intubation times were measured using a stopwatch beginning with the particular instrument entering the snout of the animal and were stopped when the connected ventilator revealed the first adequate capnography curve. Intubation attempts were aborted, when oxygen saturation dropped below 93% and animals were then mask ventilated again for 3 minutes until saturation recovered before the next try. Number of attempts until successful intubation of the trachea were counted. First pass success was noted separately. After a third unsuccessful attempt, an experienced supervisor performed the intubation.

After the intervention, the animals were ventilated, allowed to recover for 30 min and were then assigned to the primary research projects of our facility in order not to needlessly sacrifice them and adhere to ARRIVE and 3R guidelines.

### Statistics

The presented study was conducted as a pilot since no adequate data on the topic could be consulted to perform a sample size calculation. Statistical analyses were performed using the Mann-Whitney-U test via GraphPad Prism 8 software (GraphPad Software Inc., La Jolla, CA, USA) assuming a non-normal distribution. Data are presented as mean (standard deviation). *P*-values < 0.05 were considered significant.

## Results

**Fig. 2:**
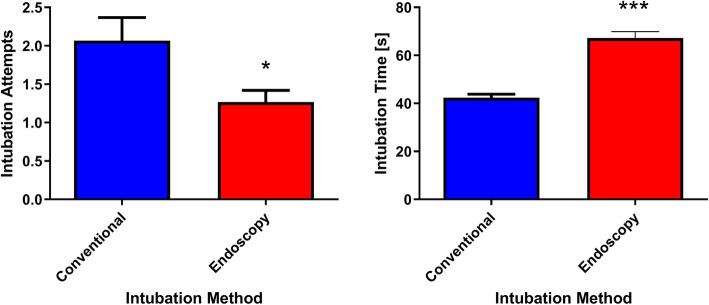
Data assessment of the trial. Number of attempts (left) and time to successful intubation (right) compared by intubation method (*n* = 15 per group). With the endoscopic technique, significantly less intubation attempts were necessary (*: *P* = 0.043). Successful video-endoscopic intubation took more time per attempt until adequate ventilation was completely established (***:*P* < 0.001) but showed less desaturation episodes. Statistical analyses were performed using the Mann-Whitney U test

All 30 animals survived the intervention in good health and no problems were encountered at any time during mask ventilation, even if tracheal intubation proved difficult. Accordingly, all animals were available for the respective primary research project without any restrictions. In the FFI group, a higher first pass success rate (75% vs. 47%) and significantly less intubation attempts were protocolled than in the CI group (1.3 ± 0.6 vs. 2.1 ± 1.3, *P* = 0.043, Fig. 2). Intervention by a supervisor to perform tracheal intubation was necessary twice in the CI group, whereas no interventions were necessary in the FFI group. Successful intubation attempts were significantly shorter in the CI group than in the FFI group (42 s ± 6 s vs. 67 s ± 10s, *P* < 0.001, Fig. 2). Intubations were performed by a total of four participants with two supervisors assessing the data and controlling the experiments. No correlations over time regarding improved performances of the individual students could be detected.

## Discussion

This study shows that FFI can be performed with a higher success rate and a better first pass success in inexperienced providers than conventional intubation by a MacIntosh blade in swine. It is the first trial to prospectively evaluate the potential benefits towards airway management using FFI in an experimental design in swine that support FFI as a reasonable method for intubation. Furthermore, a practical and efficient approach to student and resident training at the same time is described and evaluated. Our results suggest significantly less problems with the establishment of a secure airway when provided with a FFI device compared to CI. This effect proved significant in inexperienced providers as shown by the limited amount of attempts needed to place a tracheal tube. Simultaneously, first pass success rate was distinctly increased when a FFI device was used, suggesting less stress for the animal, less hypoxic episodes during induction and, subsequently, less potentially confounding factors tainting research results.

FFI required a longer intubation time when compared to CI. However, this had no clinically relevant effect. The described prolongation was most likely due to device handling, coordination with the video monitor as well as the time needed to verify tube positioning, since ventilation in our experimental setup could not be initiated before the endoscope had been removed from the tube. A longer time to intubation using endoscopic devices is common when compared to CI in humans [20, 21]. However, the time to intubation using FFI significantly improves through training and expertise [21, 22]. Furthermore, a shorter time to ventilation by FFI is possible by using respiratory adapters to facilitate ventilation during endoscopy [23].

Teaching and training health care providers to adequately apply flexible endoscopes for intubation purposes is technically challenging, expensive and time-consuming [16, 20]. Manikins as well as virtual reality simulators are reliable training methods for FFI and can facilitate skill maintenance [15, 24]. In inexperienced providers, reported times until successful FFI compass 80 s [24] up to 260 s [15], whereas success rates are cited from 50% before training and 80% after virtual reality training [24]. On the one hand, this data is based on different study designs and obviously cannot be compared with the porcine model. On the other hand, there is a trend that swine can be intubated more quickly using FFI than the airway models currently used.

One common training opportunity in clinical routine for residents is the awake FFI in patients with anticipated difficult airway. This collective is found in ear-nose-throat and maxillofacial surgery to a higher degree than in other surgical disciplines [25]. However, various national guidelines considerably differ concerning the indication of awake FFI. Moreover, local protocols and clinical routine often does not foster FFI. Thus, structured and sufficient training programs have to rely on manikins and other models as well [22]. Animal models are perceived as more realistic compared to manikins, suggesting benefits during resident training [17]. Our model could not only provide the opportunity to perform airway management training, but, in contrast to clinical approaches, more than one provider could also be trained on the same animal subsequently and repeat the procedure multiple times, offering a more efficient way to gain proficiency in device handling. Additionally, the use of a video monitor allows a supervisor to directly teach the procedure, thus potentially improving the experience even further. Single-use bronchoscopes, as shown in our trial, can also be used repeatedly to decrease material costs. Alternatively, following our own protocol, an established research facility could schedule regular short intubation trainings during the induction of their animals with the possibility to further use the animal for protocol purposes as needed afterwards. This obviously depends on experimental setups and possible confounding effects on specific studies but might be valid in some cases. Since this would decrease animal numbers by dual-use in research and education, institutional approval to respective protocol addenda should not be problematic.

Using endoscopic techniques for tracheal intubation in animals is sophisticated and rarely used, but reports in rodents [26], ruminants [27], swine [17] and more exotic animals [28] have been previously published. Interestingly, the last - and to the best of our knowledge - only published scientific use of FFI in swine was 30 years ago by Forbes et al., who not only proved feasibility, but concluded that training with a live porcine model was more realistic and had a greater clinical benefit for students and residents [17]. Unfortunately, the topic was never properly examined again, although porcine models have become an invaluable asset in translational research, especially of systemic diseases like sepsis and ARDS [29, 30] and are regularly established at university hospitals and research facilities. Most of these models usually rely on tracheal intubation [10, 12, 31], although some either resort to surgical airways, i.e. tracheostomy [32, 33], or supraglottic devices [34, 35]. However, tracheal intubation of pigs can be difficult and success depends on experience, expertise and correct preparation [12, 19]. Laryngeal anatomy of supine piglets can be challenging due to a hypermobile larynx, a long snout, deep perilaryngeal recesses and a long epiglottis that usually blocks direct access to the airway in sedated animals [9, 10]. The significantly longer oral cavity compared to humans can make it hard to visualize the epiglottis, which is long, U-shaped and often lodged on the soft palate. However, from the authors’ experience, it is usually feasible to use conventional laryngoscopes (size 4) for adequate visualization and then mobilize the lodged epiglottis with a guide rod by carefully inserting it along the soft palate in the right or left piriform recess and then perform a scooping motion to the opposite side to mobilize it. Once mobilized, as long as the animal is not repositioned, the epiglottis usually does not lodge again and intubation and visualization should be easily feasible.

Complications and mortality associated with intubation in pigs have not been comprehensively described yet, but difficult airway management is regularly mentioned [9–11]. This includes the loss of research animals during induction [13, 14] suggesting underreporting and maybe the basis of a general confounder of animal airway studies. The determined failure rate to intubate on the first attempt of over 50% in our study seems high, but retrospective analysis of our own research projects also suggests rates between 25 and 40%. The additional increase in this trial can easily be attributed to the lack of intubation experience of the performing participants. Furthermore, TTs with a diameter of 7 mm are relatively large compared to standard procedures suggested by other research groups [8, 19]. While this may have caused an increased failure rate in the CI group compared to smaller tubes, successful placement following FFI suggests a technique-related problem and not an anatomical one. Especially research protocols relying on low ventilation pressures and decreased lung stress could benefit from the possibility to use larger bore tubes. Since piglets can be easily ventilated non-invasively with a suitable mask, intubation problems rather represent a time factor than an actual hazard, delaying eventual tube placement. However, as more intensive manipulation is necessary to establish the airway during CI, increased stress and hypoxia-induced changes might affect the results of planned projects. Since no data on this cause-effect relation exist, this remains speculative. Nonetheless, our study suggests a potentially systematic benefit of a FFI approach to porcine models, which could improve scientific accuracy of experimental results. This would simultaneously decrease the animal numbers needed, thus warranting the additional economical effort of establishing the infrastructure necessary.

## Conclusion

Video-enhanced flexible fibreoptic intubation is an excellent method to safely secure the airway in swine. It can be used to provide more realistic training conditions for inexperienced providers and may simultaneously prevent airway complications, negative experimental effects and unnecessary animal losses in translational research.

## Supplementary information

**Additional file 1.** Video recorded with the Ambu aScope™ system. The video depicts laryngeal passage after epiglottis mobilisation (see Fig. 1) and endotracheal insertion of the endoscope. Note the narrow space available to pass the larynx and the anatomical angle of the trachea. Porcine tracheas tend to be longer than in humans. Additionally, the upper main bronchus usually parts above the carina (00:37). This has to be considered to prevent inadequate placement of the tracheal tube. The endoscope should not be removed until definitive visualisation of the correct tube positioning was successful.

## Data Availability

All relevant data are presented in the manuscript. Further data can be made available on reasonable request. Requests should be addressed to the corresponding author.
